# The impact of workplace violence on job burnout among Chinese correctional officers: the chain mediating effects of stress and insomnia

**DOI:** 10.1186/s12889-024-18048-1

**Published:** 2024-02-22

**Authors:** Jizhi Wang, Ying Huang, Siyuan Wang, Zheng Zhang, Yuqiong He, Xiaoping Wang, Huijuan Guo

**Affiliations:** 1 Department of Psychiatry, National Clinical Research Center for Mental Disorders, Hunan Key Laboratory of Psychiatry and Mental Health, The Second Xiangya Hospital of Central South University, National Technology Institute on Mental Disorders, Changsha, China; 2 Pingtang Compulsory Isolation Detoxification Institute in Hunan Province, Changsha, China

**Keywords:** Correctional officers, Insomnia, Job burnout, Stress, Workplace violence

## Abstract

**Background:**

The risk of workplace violence and job burnout among Chinese correctional officers is high. Stress and insomnia may influence the relationship between workplace violence and job burnout; however, this influence has been rarely studied. This study aimed to explore the effect of workplace violence on job burnout among Chinese correctional officers and to assess the contribution of stress and insomnia to this effect.

**Methods:**

In this study, the workplace violence scale, the Assens insomnia scale, the 21-item Depression Anxiety Stress Scale, and the Maslach Burnout Inventory-General Survey scale were used to assess the workplace violence, insomnia, stress, and job burnout experienced by the 472 correctional officers, respectively.

**Results:**

The results showed that (1) workplace violence was significantly and positively predictive of job burnout, (2) workplace violence affected job burnout through the mediation of stress, (3) workplace violence affected job burnout through the mediation of insomnia, and (4) stress and insomnia played fully interlocking mediating roles in the effect of workplace violence on job burnout.

**Conclusion:**

Stress and insomnia may play a full mediating role in the relationship between workplace violence and job burnout. This suggested that correctional officers may take measures to reduce stress and improve insomnia, thereby reducing their job burnout. Further research may focus on the development of effective interventions to reduce stress and improve insomnia among correctional officers.

**Supplementary Information:**

The online version contains supplementary material available at 10.1186/s12889-024-18048-1.

## Introduction

Job burnout is a state in which individuals experience emotional exhaustion, depersonalization, and a lack of personal accomplishment [1]. It affects both individuals and organizations. For individuals, job burnout may cause emotional problems such as anxiety, feelings of helplessness, irritability, alienation, apathy, aggressive behaviors, and even physical presentations [2, 3]; for organizations, it may be associated with absenteeism and reduced work efficiency [4, 5]. There has been an increase in burnout in occupational populations over the last few decades, which has become a public concern. Although burnout syndrome has been observed in all types of occupations, it tends to be more prevalent in some occupational groups, such as public safety personnel [6, 7], physicians [8–10], and teachers [11, 12]. As a specific type of public safety personnel, correctional officers face unique occupational hazards. First, prisoners are a high-risk group, and corrections officers often deal with them in their work. Second, due to the overcrowded living conditions of inmates and the possibility of discrimination and unfair treatment, the whole prison is in a tense and hostile atmosphere, and correctional officers may be exposed to a variety of threatening contingencies. Based on the above factors, Correctional officers are at high risk of experiencing violence in the workplace and could further develop into job burnout. An earlier study in Quebec found that the estimated incidence of burnout among correctional officers was 37% [13], and a study in Bulgaria reported that the incidence of burnout among correctional officers was as high as 74.5% [14]. A high incidence of burnout in correctional officers was also reported in China; for instance, Yang et al. reported that the overall burnout rate among Chinese correctional officers was 51.2% [15]. As correctional officers face serious burnout and the resultant consequences, it is necessary to explore the mechanism underlying their burnout, in order to develop intervention strategies accordingly.

Correctional officers often need to deal with hostile individuals and are potentially exposed to various threatening emergencies in their work [16]. Therefore, they are at high risk of experiencing workplace violence. Workplace violence is defined as any incident in which a person is abused, threatened, or assaulted in a work-related situation, which includes verbal abuse, threats, and physical assault [17]. Compared to individuals in other occupations, correctional officers are more frequently exposed to workplace violence [18]. A study in Australia revealed 208 incidents of violence against correctional officers in a correctional facility over a 3-year period [19]. Another study conducted in a French prison showed that more than 87% of correctional officers had experienced verbal, physical, or armed assaults [20]. Workplace violence has been found significantly associated with workplace stress, reduced job satisfaction, and increased level of burnout [21]. Previous studies in the healthcare field showed that workplace violence seriously affected workers’ self-esteem and professional identity, which eventually led to burnout [22]. Given that the experience of workplace violence is common among correctional officers and often affects their psychological and occupational health, the relationships between workplace violence and burnout among correctional officers and the underlying mechanism deserve further investigation.

Stress is a state of psychological and physiological tension experienced by an individual in response to internal or external stressors. Faced with chronic stress or single stressful life events, one’s mental well-being can be affected, leading to the development of burnout syndrome [23]. Workplace violence is one of the major sources of stress that leads to a variety of negative emotions [24]. Studies on healthcare workers found that those who had experienced workplace violence reported higher levels of stress [25, 26], indicating that workplace violence might be a strong predictor of stress [27]. A large body of evidence also suggests that excessive stress can significantly contribute to burnout. For instance, a study showed that the level of stress among healthcare workers during the COVID-19 pandemic was predictive of anxiety, depression and burnout [28]. Another study showed that burnout of physicians is often related to high work stress and poor interactions with patients and their families (e.g., verbal aggression) [29].

Insomnia is a condition in which the quality and duration of sleep are unsatisfactory for a significant period of time. It is often characterized by difficulty falling asleep, difficulty maintaining sleep, or early awakening [30]. According to relevant literature reports, the prevalence of insomnia is over 40% among correctional officers. Insomnia can cause impaired daytime functioning and a variety of negative health outcomes for correctional officers, including fatigue, daytime sleepiness, irritability and low mood, which may further affect their work performance. A variety of work-related stressors are found to be influencing factors in the development of insomnia, with one of the most prominent stressors being workplace violence. For example, studies on teachers and homecare workers found that workplace violence was associated with insomnia disorders [31, 32]. A meta-analysis also suggested a direct association between workplace violence and insomnia (OR = 2.55; 95% CI = 1.77–3.66) [33]. Furthermore, studies have found that increased stress may lead to insomnia and is a strong predictor of sleep quality [34, 35]. The relationship between insomnia and burnout has also been established by previous study [36]. It has been reported that stress-related insomnia may lead to physical and psychological exhaustion, which are symptoms of burnout. Some studies reported that employees suffering from insomnia are at an increased risk of developing burnout [37–39].

At present, numerous studies have focused on the prevalence of workplace violence or job burnout among correctional officers, with limited attention paid to the relationship between workplace violence and job burnout among correctional officers. Therefore, it is critical to focus on the topic of the impact of workplace violence on job burnout among correctional officers. At the same time, research on workplace violence and job burnout has focused primarily on healthcare workers and teachers, with limited attention paid to correctional officers, particularly in China. In addition, while some evidence suggested that stress and insomnia are related to workplace violence and job burnout, their potential interactions have not been fully elucidated. Therefore, the purpose of this study was to examine the impact of workplace violence on job burnout and to explore the role of stress and insomnia in the impact of workplace violence on job burnout. We proposed the following hypotheses:1) workplace violence may be positively associated with job burnout; 2) stress may play a mediation role in the association between workplace violence and job burnout; 3) insomnia may also play a mediation role in the association between workplace violence and job burnout; and 4) stress and insomnia play a chain mediating role between workplace violence and job burnout.

## Materials and methods

### Study participants

This cross sectional study was conducted between October 2021 and January 2022, targeting correctional officers in various provinces of China. We developed an anonymous online questionnaire using Questionnaire Star (https://www.wjx.cn), a widely used online survey platform in China. Initially, 10 correctional officers who attended an annual training program organized by the prison administration department for professional development and knowledge exchange were chosen as original deliverers. The selection criteria for these original deliverers were: (1) they volunteered to participate in the survey and were willing to recommend the questionnaire to others, and (2) they were from different provinces in China to ensure that correctional officers from different parts of China were included. Subsequently, the link to the online questionnaire was shared on popular social media platforms such as WeChat, and distributed by the original deliverers among their friends and members of WeChat groups. The first section of the questionnaire provided a concise overview of the study along with assurances regarding anonymity and confidentiality. Prior to commencing with the questionnaire, participants were required to indicate informed consent by selecting “Agree”; they also had the option to withdraw from the survey at any point by selecting “Disagree”. This study was approved by the Ethics Committee of the Second Xiangya Hospital of Central South University.

### Sample size

The sample size was determined using an online calculator (http://www.raosoft.com/samplesize.html). Considering an estimated population of 400,000 correctional officers in China, a confidence level of 95%, an accuracy level of 5%, and a response distribution of 50%, the minimum sample size was determined to be 384.

### Data filtering criteria and processes

When online data collection is complete, we perform quality control on the data. The exclusion criteria were as follows: (1) age less than 18 years and more than 60 years; (2) years of work experience greater than age; (3) age at start of participation less than 18 years; (4) scale assessments were clearly erroneous.

### Measurements

#### Workplace violence

The questionnaire includes five items: physical assault (being bitten, hit, pushed, spat on, etc.), emotional abuse (being verbally abused, humiliated, etc.), threatening intimidation (verbal, written, hand-held weapon, etc.), verbal sexual harassment (repeatedly making unwanted comments about privacy, etc.), and physical sexual harassment (unwanted touching, etc.) [40]. The response to each item is divided into 4 levels based on the frequency of occurrence, with no experience of violent event scoring 0, experience of one violent event scoring 1, experience of two or three violent events scoring 2, and experience of four or more violent events scoring 3. The workplace Violence Scale has an overall score ranging from 0 to 15, with higher scores indicating a higher frequency of violence experienced. Frequency is divided into zero frequency (0 points), low frequency (1 to 5 points), medium frequency (6 to 10 points), and high frequency (11 to 15 points). The Cronbach’s α coefficient of this scale was 0.820 in this study.

### Stress

The Depression Anxiety and Stress Scale-21 (DASS-21) [41] consists of three subscales, i.e., anxiety, depression, and stress. In this study, the stress subscale was used to assess the stress of the correctional officers. This subscale consisted of seven items, with each item assessed using a 4-point Likert scale (0 = not at all, 1 = partially applied, 2 = mostly applied, and 3 = always applied). The stress subscale, with an overall score ranging from 0 to 42, is calculated by adding the original scores of the seven items together and multiplying by 2. For this scale, a higher score indicates greater stress. In the stress scale, 15, 19 and 26 points are the cutoff values for mild, moderate and severe stress, respectively. The reliability and validity of the scale was analyzed among Chinese medical professionals and young adults, and the results showed that its reliability and validity were reliable [42, 43]. The Cronbach’s α coefficient of this scale was 0.889 in this study.

### Insomnia

The Athens Insomnia Scale (AIS) was used to assess the insomnia of the participants [44]. The scale consisted of 8 items, with the first five items assessing the quality of sleep and the last three assessing the daytime condition of the participants. All the items were rated on a 4-point Likert scale to assess the severity of insomnia. The scale has an overall score ranging from 0 to 24, with a score less than 4 indicating no insomnia, a score of 4 to 6 indicating suspected insomnia, and a score greater than 6 indicating the presence of insomnia. It has also been reported that the Cronbach’s α of this scale was 0.81 and the 2-week test-retest reliability was 0.80 in Chinese adolescents [45]. The Cronbach’s α coefficient of this scale was 0.894 in this study.

### Job burnout

Maslach Burnout Inventory-General Survey (MBI-GS) [46] was used to assess the participants’ job burnout. This 15-item scale assesses three dimensions of job burnout, i.e., emotional exhaustion, depersonalization and personal accomplishment. All the items were rated on a 7-point Likert scale (from 0 = never to 6 = every day). The total score is calculated by summing up the scores of all items, which is divided by 15 and then multiplied by 20, resulting in a modified score of 0-120. In this scale, a score below 50 indicates good job status. A score of 50–75 indicates that there is a certain degree of job burnout, which requires self-psychological adjustment. With a score of 75–100, it is recommended that the evaluator take a vacation and leave work for some time to adjust. If the score is more than 100 points, it is recommended to consult a psychologist, resign, or change jobs. The test-retest reliability (ICC = 0.71) of the scale was demonstrated acceptable in prior study [47]. The Cronbach’s α coefficient of this scale was 0.830 in this study.

### Statistical analysis

SPSS 26.0 software and the PROCESS version 4.2 were used for statistical analysis. Continuous variables were presented as mean ± standard deviation, and categorical variables were presented as the number of cases and percentage. Outcome and mediator variables were checked for multicollinearity using linear regression. If the variance inflation factor for all combinations between the result and the mediating variable is less than 5, we assume that the variables do not have multicollinearity in the model [48]. The Harman single factor method has been adopted for the common method bias test [49]. If the percentage of variance explained for factors with the first extracted feature root greater than 1 does not exceed 40%, we consider that the study does not have serious common method bias. Pearson correlation analysis was used to determine the relationship between workplace violence, stress, insomnia, and job burnout. Mediation analyse was conducted using the Model 6 of PROCESS Version 4.2 Macro for SPSS [50]. The mediating effects were determined using bootstrap 95% confidence intervals (5,000 samples), with a 95% CI that did not contain 0 indicating a significant mediating effect. For all the analyses, two-tailed *p* < 0.05 indicated statistical significance.

## Results

### General demographic information

A total of 472 correctional officers (322 males and 150 females) were included in this study. In terms of age distribution, 20.6% of the participants were 20–30 years old, 43.2% were 31–40 years old, and 29.2% were 41–50 years old. 7.0% of the participants were over 50 years old. In terms of the distribution of working time, 42.8% of the participants had been in the workforce for less than 10 years. 30.9% had been in the workforce for between 11 and 20 years. 21% had been in the workforce for between 21 and 30 years. 5.3% had been in the workforce for more than 30 years. The percentage of respondents who were single or married was close to 95%. More than 80% of the respondents had a bachelor’s degree (Table 1). The geographic distribution of the participants is presented in Table S1 of the supplementary material.

**Table 1:** General demographic information

Variable	Category	n (%)
Age (years)		
	20–30	97(20.6%)
	31–40	204(43.2%)
	41–50	138(29.2)
	51 and older	33(7.0%)
Work experience (years)		
	0–10	202(42.8%)
	11–20	146(30.9%)
	21–30	99(21.0%)
	31 and older	25(5.3%)
Gender		
	Male	322(68.2%)
	Female	150(31.8%)
Marital status		
	Unmarried	71(15.0%)
	Married	379(80.3%)
	Divorced	22(4.7%)
Level of education		
	Below bachelor’s degree	64(13.6%)
	Bachelor’s degree	380(80.5%)
	Graduate degree	28(5.9%)

M: mean, SD: standard deviation

### Multicollinearity test and common method bias test

In this study, job burnout as the dependent variable and workplace violence, stress and insomnia as the independent variables were used for linear regression analysis, and the variance inflation factor was used to assess whether there was multicollinearity in the independent variables. As the variance inflation factors for all the combinations among the outcomes and mediators were below 5, we assumed that no multicollinearity was present in the model.

In this study, the Harman single factor test was used to test the common method bias. A total of 5 factors with characteristic roots greater than 1 were extracted, among which the variance explanation percentage of the first factor was 37.29%, less than the 40% of the standard threshold value. Therefore, it could be considered that there is no serious common method bias in this study.

### The relationship between workplace violence and job burnout: a chain mediation model

There was a positive correlation between workplace violence and stress (*r* = 0.244, *p* < 0.001), insomnia (*r* = 0.264, *p* < 0.001), and job burnout (*r* = 0.189, *p* < 0.001). Stress was found positively associated with insomnia (*r* = 0.624, *p* < 0.001) and job burnout (*r* = 0.644 *p* < 0.001). Insomnia was found positively associated with job burnout (*r* = 0.545, *p* < 0.001) (Table 2).

**Table 2:** Descriptive statistics and correlation analysis of each variable

Variable	Mean ± SD	Median(25–75 percentile)	1	2	3	4
1.Workplace violence	5.91 ± 2.12	5.00(5.00–6.00)	1			
2. Stress	14.99 ± 9.30	14.00(8.00–20.00)	0.244***	1		
3. Insomnia	7.81 ± 4.77	7.00(4.25,11.00)	0.264***	0.624***	1	
4. Job burnout	51.47 ± 27.00	52.00(33.33-68.00)	0.189***	0.644***	0.545***	1

SD: standard deviation, ***p < 0.001 (two-tailed)

Model 1 showed that workplace violence was positively predictive of stress (β = 1.07, *p* < 0.001), Model 2 indicated that workplace violence (β = 0.27, *p* < 0.01) and stress (β = 0.31, *p* < 0.001) were positively predictive of insomnia, and Model 3 suggested that stress (β = 1.09, *p* < 0.001) and insomnia (β = 0.99, *p* < 0.001) were positively predictive of job burnout. However, workplace violence was not a significant predictor of job burnout (β = 0.06, *p* > 0.05) (Table 3).

**Table 3:** Sequential mediation model test

Independent Variable	Model 1 (Stress)				Model 2 (Insomnia)					Model 3 (Job burnout)				
	β	SE	t	95%CI	β	SE	t		95%CI	β	SE	t		95%CI
Workplace violence	1.07	0.20	5.44***	[0.68,1.45]	0.27	0.08	3.24**		[0.11,0.43]	0.06	0.34	0.19		[-0.61,0.74]
Stress					0.31	0.02	16.15***		[0.27,0.34]	1.09	0.10	11.28***		[0.90,1.27]
Insomnia										0.99	0.19	5.23***		[0.61,1.36]
R^2^	0.06				0.40					0.45				
F	29.65***				157.88***					126.96***				

SE: standard error, **p < 0.01 (two-tailed), ***p < 0.001 (two-tailed)

Results based on 5000 bootstrapped samples indicated that the total effect of workplace violence on job burnout was significant (βtotal = 1.81, SE = 0.43, 95% CI: LL = 0.96 to UL = 2.66, *p* < 0.001), but the direct effect was not significant (βdirect = 0.06, SE = 0.34, 95% CI: LL=-0.61 to UL = 0.74, *p* = 0.8514), suggesting that the stress and insomnia might fully mediate the relationship between workplace violence and job burnout (βindirect = 1.75, SE = 0.35, 95% CI: LL = 1.03 to UL = 2.43). Based on the pathways of mediation, it was found that the mediation effect of stress was 64.09%, the medication effect of insomnia was 14.36%, and the chain mediation effect of stress and insomnia was 17.68%. The 95% confidence interval of the above indirect effects did not include 0, indicating that the three pathways were statistically significant(Table 4).

**Table 4:** Analysis of the mediating effects of stress and insomnia

Effect	Effect value	SE	t	95%CI	Relative mediation effect
Total effect Workplace violence→job burnout	1.81	0.43	4.18***	[0.96,2.66]	
Direct effect Workplace violence→job burnout	0.06	0.34	0.19	[-0.61,0.74]	3.31%
Indirect effect	1.75	0.35		[1.03,2.43]	96.69%
Workplace violence → stress →job burnout	1.16	0.29		[0.61,1,72]	64.09%
Workplace violence → insomnia →job burnout	0.26	0.11		[0.06,0.50]	14.36%
Workplace violence → stress → insomnia →job burnout	0.32	0.10		[0.14,0.54]	17.68%

SE: standard error, ***p < 0.001(two-tailed)

## Discussion

The present study explored the effect of workplace violence on job burnout and the role of stress and insomnia in this relationship. This study found that workplace violence was positively associated with job burnout among Chinese correctional officers. Stress and insomnia were found to fully mediate the relationship between workplace violence and job burnout. Stress and insomnia were also found closely related, and both factors had a sequential mediation effect on the influence of workplace violence on job burnout.

In this study, the prevalence of burnout among correctional officers was 53.0%. Another study in China found that the burnout rate of correctional officers was 51.2% [15]. This is more consistent with the results of this study. A study in Bulgaria showed that the incidence of burnout among correctional officers was as high as 74.5% [14]. This indicated that a large percentage of correctional officers suffer from burnout. Burnout may lead to unsafe correctional practices, high absenteeism, decreased productivity, and emotional problems among correctional officers. Therefore, it is crucial to explore the factors associated with burnout among correctional officers in order to establish interventions to prevent and reduce burnout. Next, this study would discuss the effects of workplace violence, stress, and sleep on burnout, as well as the mediating role of stress and sleep in the effects of workplace violence on burnout.

This study found that workplace violence was a positive predictor of job burnout, with the correctional officers who experienced more workplace violence having a higher level of job burnout; conversely, those who experienced less workplace violence reported a lower level of job burnout. This is consistent with the findings of existing studies [51]. In prisons, correctional officers are required to perform a wide variety of job tasks, including ensuring the safety of inmates, coworkers, and themselves. In addition, the primary duties of correctional officers involve correctional actions against some inmates against their will; although most of the inmates are compliant, the officers may also be faced with resistant and disruptive behaviors [52], which result in potential and omnipresent conflict and even violence in their work environment [53]. Therefore, correctional officers are at a high risk of experiencing workplace violence [54, 55]. A study examining the prevalence of workplace violence among correctional officers in the United States from 1999 to 2008 found that 113 correctional officers were killed and 125,200 were injured, with assault and violence (38%) being the main events leading to non-fatal injuries [56]. A study on workplace violence experienced by correctional officers in Brazilian female prisons found that the prevalence of their experiencing at least one violent event (physical, psychological, sexual and moral violence) was 28.4% [57]. Considering that women are less aggressive than men, it could be seen that the prevalence of workplace violence among correctional officers in Brazil is higher than 28.4%. In this study, the prevalence of workplace violence (physical assault, emotional abuse, threats of intimidation, verbal sexual harassment, and physical sexual harassment) experienced by correctional officers was 27%. As can be seen from the above studies, the prevalence of workplace violence among correctional officers in the United States and Brazil is higher than the present study. This may be related to differences in prison management systems in different countries, types of prisoners and other factors. Workplace violence may increase stress on corrections officers. Chronic stress in the workplace may lead to fatigue and a sense of being overwhelmed, which may result in their excessive use of psychological and emotional resources to cope with the violent events. Eventually, the correctional officers may feel traumatized with a reduced sense of trust and security and increased negative feelings and resistance, which results in job burnout over time.

Stress plays a partial mediating role in the relationship between workplace violence and job burnout. The present study suggested that greater workplace violence and stress were associated with higher levels of job burnout among Chinese correctional officers. The threat-stress model posits that a threatening event or attack may trigger physical and psychological stress responses in an individual. Workplace violence, which threatens people’s safety, may trigger individuals’ defense mechanisms, resulting in their tension, anxiety, and inability to cope. Therefore, correctional officers exposed to chronic stress may feel exhausted both physically and psychologically, which can eventually lead to job burnout.

In this study, we also found that insomnia partially mediated the relationship between workplace violence and job burnout. Sleep is an important process for physical and psychological recovery, and sleep deprivation can affect the restoration of one’s physical and mental energy and resources, leading to poor concentration, slowed reactions, emotional instability, and anxiety [58]. The sleep status of correctional officers can be affected by exposure to workplace violence. A study found that the prevalence of severe sleep disorders reached 55.3% among 374 correctional officers [59]. Furthermore, using the Pittsburg Sleep Quality Index, a study involving 355 correctional officers in Washington State found a prevalence of insomnia of 44.8% [38]. In a study of 376 Indonesian and 288 Polish correctional officers, insomnia was assessed using the Assens Insomnia Scale. The results showed that 43.4% of the Polish correctional officers and 26.1% of the Indonesian correctional officers showed early symptoms of insomnia [60]. The above studies indicated that a large percentage of correctional officers showed insomnia symptoms. Chronic insomnia may lead to a drain on energy and resources, which ultimately leads to job burnout.

This study found that stress and insomnia were closely related, and there was a chain mediating effect between the two in the influence of workplace violence on job burnout, i.e., if correctional officers who were exposed to workplace violence experienced greater stress, they might also experience a higher level of insomnia and job burnout. Workplace violence per se can lead to increased stress among correctional officers. Biologically, a high level of stress can lead to the release of hormones such as adrenaline and cortisol, which activate the nervous system and the body’s stress response [61]. This biological response may keep an individual alert but make it difficult for them to relax and fall asleep [62]. In addition, individuals in stressful environments often experience fear and anxiety, which can lead to circular thinking and anxiety at night, resulting in difficulty falling asleep and poor sleep quality [63]. Therefore, insomnia has become one of the influencing factors of job burnout among correctional officers.

### Limitations and future directions

There are several limitations in this study. First, this study was cross-sectional, which precluded us from exploring causal relationships. Second, this study used a non-probability sampling method to recruit participants, which might have limited the generalizability of the findings. Future studies may use the random sampling method to increase generalizability to larger populations. Longitudinal studies are also needed to examine the potential causal relationship between workplace violence, stress, sleep, and job burnout.

## Conclusion

The present study demonstrates that workplace violence has effects on the job burnout of correctional officers. Meanwhile, stress and insomnia play a full mediating role in the association between workplace violence and job burnout. Our findings enrich our understanding of job burnout in correctional officers and how to reduce it. This study provides a new possibility for the prevention of job burnout in correctional officers, that is, to provide relevant facilities or psychological interventions to prevent and reduce stress and insomnia in correctional officers. In addition, more similar studies in different populations are needed to confirm this idea in the future.

### Electronic supplementary material

Below is the link to the electronic supplementary material.

Additional file 1: Table S1. The geographic distribution of the participants.

## Data Availability

All data for this research article is available and can be accessed from the corresponding author at any time.
