# Supplementary material for: The impact of workplace violence on job burnout among Chinese correctional officers: the chain mediating effects of stress and insomnia
Source: BMC Public Health. 2024 Feb 22;24:566. doi: 10.1186/s12889-024-18048-1 (PMC10885376; doi:10.1186/s12889-024-18048-1)
Supplement: Supplementary file 1 — Additional file 1: Table S1. The geographic distribution of the participants. [file 12889_2024_18048_MOESM1_ESM.docx]

Table S1. Geographic distribution and age distribution of participants

| **Variable** | **Category** | **Number** | **Age** |
| --- | --- | --- | --- |
| geographic distribution |  |  | **mean±SD** |
|  | Hunan province | 294 | 37.49±8.14 |
|  | Henan province | 17 | 36.00±7.24 |
|  | Inner Mongolia  Autonomous Region | 15 | 40.47±8.42 |
|  | Fujian province | 12 | 44.25±10.08 |
|  | Hubei province | 12 | 35.75±10.45 |
|  | Shandong province | 11 | 36.91±11.42 |
|  | Qinghai province | 9 | 32.56±5.15 |
|  | Hebei province | 9 | 37.11±7.22 |
|  | Shanxi province | 8 | 35.75±5.18 |
|  | Beijing | 8 | 37.88±10.59 |
|  | Jiangsu province | 8 | 37.88±7.53 |
|  | Jiangxi province | 8 | 36.75±5.55 |
|  | The other 17 provinces | 61 | 38.47±6.89 |
